# Supplementary material for: DrugGym: A testbed for the economics of autonomous drug discovery
Source: bioRxiv. 2024 Jun 2:2024.05.28.596296. Preprint. [Version 1] doi: 10.1101/2024.05.28.596296 (PMC11160604; doi:10.1101/2024.05.28.596296)
Supplement: Supplement 1 [file NIHPP2024.05.28.596296v1-supplement-1.pdf]

bioRxiv preprint doi: https://doi.org/10.1101/2024.05.28.596296; this version posted June 2, 2024. The copyright holder for this preprint (which was not certified by peer review) is the author/funder, who has granted bioRxiv a license to display the preprint in perpetuity. It is made available under aCC-BY 4.0 International license.

Appendix: DrugGym

Michael Retchin* **(ORCID: 0000-0001-9822-8318)** [1], **Yuanqing Wang (ORCID: 0000-0003-4403-2015)** [2, 3],
Kenichiro Takaba (ORCID: 0000-0002-2481-8830) [2, 4],
John D. Chodera* **(ORCID: 0000-0003-0542-119X)** [1,2]

[1]Tri-Institutional PhD Program in Computational Biology and Medicine, Weill Cornell Medical College, Cornell University, New York, NY 10065; [2]Computational and Systems Biology Program, Sloan Kettering Institute, Memorial Sloan Kettering Cancer Center, New York, NY 10065; [3]Simons Center for Computational Chemistry and Center for Data Science, New York University, New York, NY 10004; [4]Pharmaceutical Research Center, Advanced Drug Discovery, Asahi Kasei Pharma Corporation, Shizuoka 410-2321, Japan

***For correspondence:**
michael.retchin@choderalab.org (MR); john.chodera@choderalab.org (JDC)

Detailed methods

DrugGym

DrugGym (www.drug-gym.org), our framework for modeling the stochastic process of drug discovery, is implemented in **Python** (version: 3.10 and above). It extends the popular **Gymnasium** library (version: 1.0.0) [102] for testing reinforcement learning (RL) algorithms.

DrugGym implements a *DrugAgent* and a *DrugEnvironment*. An experiment is run with a predefined budget, defined in terms of number of molecules made, total monetary cost, the number of DMTA cycles, or all of these. At each step, the DrugAgent receives observations and emits actions, while the DrugEnvironment does the opposite. The observations that the DrugAgent receives are the current library of molecules maintained inside the DrugEnvironment. This is an array: each row represents an individual molecule, and the columns represent all the information that is known about the molecule, such as its SMILES and other properties that may have been recorded (predictive scores and measurements). There are also columns for the current status of the molecule, which may be set to *Designed*, *Scored*, *Made*, or *Tested*, as well as the timestep when the transition was made to its current status. A molecule is Designed if it has just been ideated but no further action has been taken, Scored when the molecule is scored by every surrogate model available (with non-zero model error, called *NoisyOracles*), Made if the molecule is recorded as having been synthesized, and Tested when the molecule is run through every surrogate model available (with zero model error, called *Oracles*). Individual cells in the DrugEnvironment's library (equivalent to the DrugAgent's observations) may be empty, for example if a molecule has just been Designed but no additional actions have been taken.

After every step, the DrugEnvironment is responsible for ending or continuing the episode. Using its *Policy* (defined below), it assesses every molecule that has been fully Tested. If any molecule returns a utility of 1.0, the episode is ended ("terminated," in the parlance of the Gymnasium library). An episode will also finish if the budget is exhausted ("truncated").

DrugAgent

DrugAgents are initialized with a Policy object, an ExploratoryStrategy object, and a sequence of action templates.

Each action template is a dictionary containing the name of the action to be undertaken (e.g., "design," "Noisy ABL1 pIC50 docking," "make," "ABL1 pIC50 docking"), any parameters to associate with that assay (e.g., the number of steps to run the simulation or the ideation temperature parameter for the design step), and a batch size, which is an integer that will determine the number of molecules with which the action will be performed. The intent of this action sequence is to mimic choices that a medicinal chemist might make in a typical ADME assay cascade [53]. The DrugAgent generates an action based on the information currently

bioRxiv preprint doi: https://doi.org/10.1101/2024.05.28.596296; this version posted June 2, 2024. The copyright holder for this preprint (which was not certified by peer review) is the author/funder, who has granted bioRxiv a license to display the preprint in perpetuity. It is made available under aCC-BY 4.0 International license.

available in its actions. As it does, it replaces the batch size with an appropriate number of indices, which are associated with rows in the DrugEnvironment's library. These are the chosen compounds on which the action will be performed.

To enforce the DMTA cycle progression, the DrugAgent filters observations depending on the action that is anticipated in the next step, outlined below:

Table S 2. Actions and Filter Criteria

Action Type	Filter Criteria	Reasoning
Design	Scored OR Tested	Do not ideate analogs of molecules for which no information is known
Make	Scored OR Designed	Do not order a synthesis of a molecule that has already been synthesized
Type starting with "Noisy" (i.e., Score step)	Designed only	Do not score molecules that have already been scored, made, and tested
All other actions (i.e., Test step)	Made only	Molecules that have not been synthesized cannot be tested

The DrugAgent selects these molecules using its Policy and ExploratoryStrategy. The Policy computes a "utility" for each molecule using all available information (scores, given by NoisyOracles; and measurements, given by Oracles). Briefly, our standard Policy employs a hybrid sort that includes non-dominated sorting [71] into Pareto fronts and ranking by weighted average within fronts. The Policy is described in more detail below. Given a ranking of these utilities, the ExploratoryStrategy returns a final selection. The purpose of the ExploratoryStrategy is to balance exploiting molecules that appear to be maximally valuable in the present step with the option to explore unknown but potentially promising chemical space. While our experiments do not include a step for updating our models, nor do we use probabilistic predictions as in Bayesian optimization, all of our scores and tests are associated with irreducible error, so striking a balance is likely to improve search efficiency. Here, we use an ϵ-greedy strategy, which is a simple and interpretable option for parameterizing the tradeoff between exploitation and exploration [73].

DrugEnvironment

DrugEnvironments are initialized with an integer budget, Policy object, list of assays (Oracles) and surrogate predictive models (NoisyOracles), and a Designer, responsible for taking molecules and returning molecules nearby in chemical space. We describe the Designer in-depth below. The DrugEnvironment parses actions from the DrugAgent. It ensures that the DMTA progression is respected by asserting conformity to the action filter criteria outlined above. As it performs the action, it annotates the value for the corresponding row in its library and also annotates a timestamp of the action. We use this timestamp for later accounting of program lengths and costs.

The choice of Policy, which is how the environment assigns utility to the molecules in its library, is slightly different from that of the DrugAgent. The DrugAgent uses a hybrid sort that incorporates non-dominated sorting, because it must rank molecules by considering tradeoffs of several competing objectives. However, the DrugEnvironment is not ranking but rather reporting the maximum utility observed. Therefore, the Policy of the environment returns a simple weighted average from the utility of each objective (this is identical to the second step of the hybrid sort).

After every step, the DrugEnvironment returns observations (its library), reward (the maximum utility observed for any individual molecule in its library for which all measurements are available), termination state (met objectives), and truncation state (exhausted budget). If either termination or truncation states are set to 1, the episode ends. This construction implements the Gymnasium API.

bioRxiv preprint doi: https://doi.org/10.1101/2024.05.28.596296; this version posted June 2, 2024. The copyright holder for this preprint (which was not certified by peer review) is the author/funder, who has granted bioRxiv a license to display the preprint in perpetuity. It is made available under aCC-BY 4.0 International license.

Designer

Our Designer is intended to mimic standard routines of medicinal chemistry. Given a starting molecule (identified by a SMILES) and its synthesis route, we run one of two routines, inspired by previously reported ideation strategies for hit-to-lead candidates [115].

Replace. Given a fixed route (provided by the original molecule), we replace one or more reactants. The "new" reactant that replaces the old comes from the **Enamine** Global Stock (version June 30, 2023) [54], and the search criteria for the new reactant is the sum of Tanimoto similarity based on ECFP4/1 1024 bit fingerprints computed by **OpenBabel** (version: 3.1.1) and a size similarity score based on the number of heavy atoms in the molecules, computed by **RDKit** (version: 2024.03.1). We consider size explicitly during this search because Tanimoto similarity alone does not take it into account. We found that it will rank a building block with several identical repeats of patterns from the original reactant (e.g., a heterocycle) over one that is off by a single atom. We believe this implementation is closer to the meaning of similarity. The ranking over all of the building blocks is given by **chemfp** (version: 4.1) [116].

We re-weight the ranking with a Boltzmann distribution parameterized by an ideation temperature T:

$$p_i \propto \exp\left(-\frac{s_i}{kT}\right)$$

where p_i is the new probability assignment for the ith building block, s_i is the similarity score compared to the original molecule for the same building block, and kT is the product of the Boltzmann constant with the ideation temperature. To obtain the new ordering, building blocks are sampled without replacement from a multinomial distribution (**PyTorch** version: 2.3.0) using the values of p_i as weights. Higher temperatures are more likely to select building blocks that are further down the ranking; lower temperatures conform to the original order. Hence, high temperatures are more adventurous. An additional parameter is the number of reactants that may be replaced as part of any given sampled molecule, compared to the original synthesis route. Since throughput is bottlenecked by reading the building blocks from an SDF on disk, we perform the replacements using lazy generators that minimize disk I/O. For multi-step reactions, we generate reactants at reaction-time, working from the "leaves" of the reaction tree toward the product. Reactions are run forward (using **RDKit** version: 2024.03.1) until sufficient products are produced. Upon failure due to reactant incompatibility, the loop continues to the next set of reactants until a sufficient number of products are enumerated.

Grow. The original molecule is used as one of the reactants, with the other being a randomly chosen building block. To avoid oversized products, we limit the size of the chosen building block to 10 heavy atoms. Since the appropriate reaction is unknown ahead of time, every reaction in the user-defined reaction repertoire is attempted in every loop. If none are compatible, the loop continues with the next randomly-chosen building blocks. The Grow routine in particular is necessary to approach the size needed for achieving drug-likeness [117].

All reaction products are kekulized in **RDKit** (version: 2024.03.1) and each resulting SMILES is recorded in canonical form. Other general features of the Designer include its cache, maintained so that it avoids generating the same molecule again, and an optional flag that protects non-reactive atoms from participating in subsequent reactions. This feature, and another that enforces a strict substructure match in building blocks, can be used to restrict the chemical space that can be enumerated. By default, all permutations of reactants are attempted with a reaction before moving on to the next set. Our reactions are based on SMIRKS patterns from **SmilesClickChem** (version: 1.0.1). We found that the order of the reactions matters, since some reactions tend to be more generally compatible than others. To estimate the general compatibility, we tested each reaction for compatibility against 10,000 samples of Enamine building block pairs. We reordered the reactions based on these estimates.

We are not the first to propose a synthetic enumeration system like this [118], but we have made it very efficient (**Supplementary Figure 18**).

bioRxiv preprint doi: https://doi.org/10.1101/2024.05.28.596296; this version posted June 2, 2024. The copyright holder for this preprint (which was not certified by peer review) is the author/funder, who has granted bioRxiv a license to display the preprint in perpetuity. It is made available under aCC-BY 4.0 International license.

Oracle

Oracle functions in DrugGym are intended to be realistic surrogates of real drug discovery assays. They receive molecules and return a value for each molecule. We also implement a NoisyOracle to represent predictive models, and we call these predictive models. These wrap Oracles with an additive Gaussian error, which can be parameterized with location and spread parameters. Here, we only modify the spread, so our error distributions are unbiased relative to the original Oracle values. We now describe the different Oracle functions. [1]

ABL1 pIC50. We use docking to mimic realistic structure-activity relationships that possess activity cliffs, which frequently frustrated real drug discovery programs [45]. For ligand preparation, we employ **RDKit** (default settings, version: 2024.03.2) for protonation and conformer generation, and **Meeko** (version: 0.5.0) for computing charges (default settings except `rigid_macrocycles=True`). We do not perform target preparation ourselves, instead using a prepped ABL1 pocket fragment given in **DOCKSTRING** (version: 0.3.2) [119]. We adopt their pocket center, which is `(15.851, 14.647, 3.904)`. For docking, we make use of **uni-dock** (version: 1.1.2 with conda installation), a GPU-accelerated implementation of **Autodock Vina** [56, 120]. We use the "detailed" setting, which sets exhaustiveness to `512` and max-step to `40`. All other settings use defaults. **Uni-dock** returns a docking score in terms of ΔG, but we wish to interpret results in terms of pIC50. Therefore, we implemented a conversion to pIC50, which involves multiplying the docking score (which is in terms of ΔG kcal/mol) by a coefficient.

To derive that coefficient, we note that $K_i = c_0 e^{-\Delta G/(k_B T)}$, where c_0 is a reference concentration (here, 1 M); we assume the enzyme obeys Michaelis-Menten kinetics, allowing us to relate the half maximal inhibitory concentration (IC50) to the inhibition constant K_i using the Cheng-Prusoff equation:

$$\mathrm{IC}_{50} = \left(1 + \frac{[\mathrm{S}]}{K_m}\right) \cdot K_i \tag{1}$$

We assume that our assay has [S] $<< K_m$, such that IC50 $\approx K_i$). The definition of pIC50 is $-\log_{10}(\mathrm{IC50}/c_0)$. Then,

$$\begin{aligned} \mathrm{pIC}_{50} &= -\log_{10}(\mathrm{IC}_{50}/c_0) && (2)\\ &\approx -\log_{10}(K_i/c_0) && (3)\\ &= -\log_{10}\left(e^{-\Delta G/(k_B T)}\right) && (4)\\ &= \frac{\Delta G}{k_B T}\log_{10} e && (5) \end{aligned}$$

At room temperature ($T = 300$ Kelvin), $k_B T \approx 0.596$ kcal/mol.

Log S. We trained a **CatBoost** model (version: 1.2.5) [121] on **AqSolDB** [61]. Like ChemProp-RDKit [122], we generate input features for regression using RDKit, run with multiprocessing via **scikit-mol** (version: v0.2.0) [123]. These features are: `ExactMolWt,FpDensityMorgan1,FpDensityMorgan2,FpDensityMorgan3,HeavyAtomMolWt,MaxAbsPartialCharge,MaxAbsPartialCharge,MinAbsPartialCharge,MinPartialCharge,MolWt,NumRadicalElectrons,NumValenceElectrons,MolLogP,FractionCSP3,HeavyAtomCount,NHOHCount,NOCount,NumAliphaticCarbocycles,NumAliphaticHeterocycles,NumAliphaticRings,NumAromaticCarbocycles,NumAromaticHeterocycles,NumAromaticRings,NumHAcceptors,NumHDonors,NumHeteroatoms,NumRotatableBonds,NumSaturatedCarbocycles,NumSaturatedHeterocycles,NumSaturatedRings,RingCount`. Using a uniform sampling strategy, we split these data into 80% train and 20% test, and our model recorded 0.784 MAE on the test set (**Supplementary Figure** 13). Our results compare favorably to the current leaderboard on the Therapeutics Data Commons [36].

Log P. We use **RDKit**'s MolLogP descriptor, which is an implementation of Crippen's method [58].

[1] Our convention is to prefix "Noisy" to the names of NoisyOracles (that of "ABL1 pIC50" would be "Noisy ABL1 pIC50"), and we use the term "assay" to refer to the task they share with their corresponding Oracle (in this case, "ABL1 pIC50").

bioRxiv preprint doi: https://doi.org/10.1101/2024.05.28.596296; this version posted June 2, 2024. The copyright holder for this preprint (which was not certified by peer review) is the author/funder, who has granted bioRxiv a license to display the preprint in perpetuity. It is made available under aCC-BY 4.0 International license.

Utility function

The utility function maps from the values received from an Oracle to $(-\infty, 1]$, expressing the relative benefit that can be gained from selecting a molecule with that value. This mapping is determined by a user-defined target candidate profile (TCP), which is a set of threshold pairs (an upper and lower value to signify acceptable and ideal ranges). For all experiments, we use a challenging but not overly difficult TCP that includes ABL1 pIC50, log S, and log P (**Supplementary Table** 3, reproduced from the **Introduction**).

Table S 3. Target candidate profile (TCP) objectives used in this work. Associated methods used here for computing corresponding Oracles in DrugGym are noted.

Objective	Units	Oracle Method	Dynamic Range	Thresholds	
				Acceptable	*Ideal*
**Affinity**	pIC50	Docking [56]	[3, 11] [57]	[8, ∞)	[9, ∞)
**Lipophilicity**	Log P	RDKit [58]	[-1, 8] [59]	[0, 4]	[0, 3]
**Solubility**	Log S	Gradient Boosted Tree [60]	[-8, 1] [61]	[-4, 0]	[-3, 0]

The corresponding oracle classes are DockingOracle, CatBoostOracle, and RDKitOracle, described above.

Given the TCP, we define a piecewise multi-dimensional utility function as follows:

$$\text{net utility} = \left(\sum_{j,k} w_k \cdot \text{utility}_k(x_j) \right) - \text{costs} \tag{6}$$

$$\text{utility}(x) = \begin{cases} 1 - \left(\frac{|a_0 - x|}{i_0 - a_0} + 1 \right)^2 & \text{if } x < a_0 \\ 1 - \frac{i_0 - x}{i_0 - a_0} & \text{if } a_0 \leq x < i_0 \\ 1 & \text{if } i_0 \leq x \leq i_1 \\ 1 - \frac{x - i_1}{a_1 - i_1} & \text{if } i_1 < x \leq a_1 \\ 1 - \left(\frac{|x - a_1|}{a_1 - i_1} + 1 \right)^2 & \text{if } x > a_1 \end{cases} \tag{7}$$

This functional form was chosen because it implies that any value within the ideal range is equally valuable, and that no molecule can be more valued than those within the ideal range; that there is a monotonic increase in value for molecules that fall between the acceptable and ideal ranges; and that values whose properties fall outside of the acceptable ranges are proportionally penalized. In this case, with a quadratic penalty common to constrained optimization problems [68]. Our goal is to find a molecule that maximizes utility.

Policy

The Policy is designed to return a scalar that can be used by the DrugAgent to select molecules. We draw a distinction between values returned by Oracles ("measurements") and those returned by NoisyOracles ("scores"). By construction of the action filtering criteria discussed in DrugAgent, we will have scores for any molecule that has measurements, but not the converse. Therefore, we implemented a graceful fallback, which uses measurements when they are available, but otherwise uses scores in their place. To implement a well-defined metric space, molecules can only be compared if the resulting values are complete, by which we mean that every "assay" has an associated score or measurement.

To compose utilities across multiple objectives, we use one of two methods. The first and simpler is weighted average (alternatives here include min, max, sum, and product, compared in **Supplementary Figure** 18). The weights are user-defined. Here, we use 0.8 for pIC50, 0.1 for log S, and 0.1 for log P, and we do not deviate from these in any of our experiments. It is not necessary that they add up to 1. We chose these weights to influence the optimization, but different weights could easily be chosen, or even optimized; a weights schedule that changes according to traditional assay tiers is also possible. The weighted average sort is used to obtain the current reward in the DrugEnvironment. We also implement a hybrid sort, which

bioRxiv preprint doi: https://doi.org/10.1101/2024.05.28.596296; this version posted June 2, 2024. The copyright holder for this preprint (which was not certified by peer review) is the author/funder, who has granted bioRxiv a license to display the preprint in perpetuity. It is made available under aCC-BY 4.0 International license.

is used in the DrugAgent. Our hybrid sort uses NSGA-II (**pymoo** version: 0.6.1) to define non-dominated fronts. We use the same weighted average as before to break the tie within fronts. These rankings are composed using `numpy.lexsort` (**NumPy** version: 1.26.4). We chose this criteria because it satisfies Pareto optimality [69] and minimizes deviation from the ideal range of the TCP.

After several rounds of DMTA, we will have some molecules with complete measurements, and others that have only been scored. Which molecules should serve as the basis for designing analogs in subsequent DMTA cycles? One rule is to ideate around tested molecules only. However, this severely limits the search space and may get trapped in local minima, especially in the early part of campaigns. We sought a procedure for selecting from scored and tested molecules on equal footing. While we expect the Gaussian error of our predictions to be homoscedastic, this is not true under selection. In that case, the ranking of observations considers both the underlying "true" values and the noise. Hence, the distribution of this subset will systematically overestimate the true value with high probability (**Supplementary Figure** 17). To address this, we employ a simple linear correction that uses available measurements and corresponding predictive scores from molecules that have been tested. At every time step, we fit an OLS regressor (**statsmodels** version: 0.14.1) that maps from predictions to actual measurements. This doesn't improve predictive power, but it does rescale scores so that predictions and actual measurements can be directly compared in the selection step. We employ the OLS correction only after 30 molecules have been tested, so sample statistics have approximately converged. The correction is applied only to scored molecules (not actual measurements), and these corrected scores are used in the utility function transformation.

ExploratoryStrategy

Given the utility predicted by the Policy, the DrugAgent must choose the indices of molecules to progress into the next DMTA cycle action. We call this function the ExploratoryStrategy. We use the classic ϵ-greedy method, because it is a competitive method that trades off exploration and exploitation in an interpretable fashion [73]. Another simple alternative would be Boltzmann exploration [124], but other ExploratoryStrategies could be adopted as part of a more sophisticated DrugAgent architecture employing neural networks and search algorithms.

Experimental Defaults

Hit selection. The standard setting of the experiments begins when 5 starting hits are selected from the SDF of **Enamine**'s DSI-poised Library (version: 2 March 2021) [125]. This selection is restricted to the library subset that is compatible with the two-reactant, one-step reaction repertoire from **SMILESClickChem** (version: 1.0.1) [55]. Reaction compatibility is determined by non-zero product yields from permuting the reactant ordering with forward synthesis in **RDKit** (version: 2024.03.1) [126].

Action sequence. A sequence of action templates determines what action will be generated next by the DrugAgent.

Table S 4. Baseline action sequence for all experiments. Every product generated in the Design step is scored in the Score step that immediately follows.

Sequence	Action Type	Batch Size	Parameters
1	Design (Replace)	8	Products: 5, Temperature: 0.16
2	Score	40	Noisy ABL1 pIC50, Noisy Log P, Noisy Log S
3	Design (Grow)	8	Products: 5
4	Score	40	Noisy ABL1 pIC50, Noisy Log P, Noisy Log S
5	Make	8	
6	Test	8	ABL1 pIC50, Log P, Log S

Every product generated in the Design step is scored in the Score step that immediately follows it. The effect of this default is that 10 compounds are scored for every one that is made and tested. Our ϵ-greedy uses

bioRxiv preprint doi: https://doi.org/10.1101/2024.05.28.596296; this version posted June 2, 2024. The copyright holder for this preprint (which was not certified by peer review) is the author/funder, who has granted bioRxiv a license to display the preprint in perpetuity. It is made available under aCC-BY 4.0 International license.

$\epsilon = 0.2$, meaning that 20% of the time, a compound was chosen at random (without replacement) rather than according to the utility provided by the Policy.

Chemical properties

We ran 100 trials using the default parameters outlined above (with $\sigma = 1.0$ used in the NoisyOracles surrogate models of each of our TCP objectives). Due to measurement error, stochasticity in decision-making, and the varying quality of starting hits, programs complete at different times. To make an appropriate comparison across compounds at the beginning, middle, and end of campaigns, we create a normalized measure of campaign progress. Following Beckers et al. [79], we measure progress as the percentage of compounds that have been "made" in the simulation. We use progress percentage to study longitudinal impacts of different ideation and selection strategies.

To generate **Figure 4B**, we first compute cumulative progress percentage assignments. That is, for each progress percentile, we gather all the molecules that have had this progress percentage or lower (i.e., 100% cumulative progress should be very smooth, since it contains *all* molecules in the series). We pool this analysis across every trial. Since we are interested in the right tail of the distribution, we compute the cumulative distribution (CDF) for molecules that can be found at a given ABL1 pIC50 value or *greater*, stratified by the cumulative progress percentage just described.

Figure 4C plots the *average* of various computed properties on the same dataset across the progress percentage (not cumulative), with 99% bootstrapped confidence intervals. These properties include: ABL1 pIC50, Log P, Log S, HeavyAtomCount, RingCount, FractionCSP3, LiPE, LE, and QED. The first three were computed using our DrugGym Oracles as described before. The rest were computed using **RDKit** (version: 2024.03.1).

Figure 4C visualizes the vertices and edges of the design lineage graph for molecules that were made within a single representative trial. We constructed the graph using **networkx** (version: 3.3) [127] and used the Reingold-Tilford algorithm implemented in **python-igraph** (version 0.11.5) [128] to generate coordinates. Edges are cubic Bezier curves. The color intensity is given by the utility function for the corresponding TCP measures from the compounds.

Chemical structures

In **Figure 5A**, the same normalization procedure as described in the previous section was used to compute progress percentage. We used the same 100 trials with $\sigma = 1.0$ for the NoisyOracles. We pooled all the Made molecules and segmented them by progress percentage. We then sampled molecules randomly and visualized them using **RDKit** (version: 2024.03.1).

The synthetic route in **Figure 5B** was created by selecting one of the best-performing molecules across all of our simulations and using the `dgym.molecule.Molecule.dump` method to obtain its synthetic route (referring to "dumping" or serializing a data model). The synthetic route is preserved from when the molecule was generated by the Designer. It includes SMILES, building block identifiers from **Enamine**, and reactions. An example synthetic route dump:


```
{
    "product": "CC(NC=O)C(=O)N(C(=O)c1cc(-c2cc(O)cc3c2OCCO3)ccc1Cl)C1CC1",
    "reaction": "23_Ester_and_Amine",
    "reactants": [
        {
            "product": "O=C(NC1CC1)c1cc(-c2cc(O)cc3c2OCCO3)ccc1Cl",
            "reaction": "79_Stille",
            "reactants": [
                {
                    "product": "O=C(NC1CC1)c1cc(Br)ccc1Cl"
                },
```

bioRxiv preprint doi: https://doi.org/10.1101/2024.05.28.596296; this version posted June 2, 2024. The copyright holder for this preprint (which was not certified by peer review) is the author/funder, who has granted bioRxiv a license to display the preprint in perpetuity. It is made available under aCC-BY 4.0 International license.

```
                {
                    "product": "Oc1cc(Br)c2c(c1)OCCO2"
                }
            ]
        },
        {
            "product": "CC1NC(=O)COC1=O"
        }
    ]
}
```

We visualized these molecules in **RDKit** (version: 2024.03.1).

The molecules in **Figures** 6 and 7 were also visualized in RDKit, and their properties were computed using DrugGym Oracles as described previously. The DMTA cycle value is that step assigned to the molecule when it was synthesized (assigned Made status).

Ideation creativity

Figure 8**A** is a conceptual mockup based on a blog from Greg Landrum [129], intended to illustrate the Designer routines described earlier.

Figure 8**B** was created by repeated use of the DrugGym Designer: first, two **Enamine** building blocks were chosen at random. Then, all the reactions from **SmilesClickChem** were tested for compatibility with these reactants. The first to prove compatible was used to construct a product. This process was repeated with one more building block (using the Designer's Grow routine). Finally, we apply the Designer's Replace mode with different setting of ideation temperature `0.00, 0.04, 0.16`, and `1.00` across 1, 2, and 3 reactant replacements. The resulting products were visualized using **RDKit** (version: 2024.03.1).

For **Figure** 8**C**, we use a batch size of 24 rather than the usual batch size of 8. However, this batch size was used across all the trials and experimental settings shown in this figure. We use **lifelines** (version: 0.27.8) to compute a Kaplan–Meier estimator of the probability that an experiment had not yet terminated successfully by a given cost, such as the number of molecules made. We use this estimator because it was designed to be robust to right-censorship, as can occur with very long-running simulations like ours. We compute the Kaplan-Meier estimator for trials performed with escalating ideation temperatures: `0.0`, `0.02`, `0.04`, `0.08`, `0.16`, `0.32`, and `0.64`. In addition, each of these ideation temperature settings was tested with a different maximum of reactant replacements: 1, 2, or all reactants in the synthetic route. To derive the plot shown, we find the Kaplan-Meier estimate of success rate corresponding to a 3200 molecule budget. These success rates represent the proportion of trials that are expected to succeed within 3200 molecules *or fewer*. The 68% confidence interval calculated by Greenwood's Exponential formula is represented by the shaded region.

The same analysis is extended in **Figure** 8**D**, except that the targeted quantity is 0.95 success rate. We use the Kaplan-Meier estimator at or above 0.95 success rate to find costs. These should be interpreted as the "prices" necessary to provide guarantees for given probabilities of success. They are not estimates of the *average* cost.

Model error

In this experiment, we held all variables constant except for the σ associated with the NoisyOracles (ideation temperature was set to 0.16). We ramped σ from `0.0` to `2.0` in increments of `0.5`. As before, all experimental settings were conducted with 100 replicates.

Figures 10**C** and **D** are identical to the two ideation creativity experiments described just above, except that the exogenous variable is model error (σ).

bioRxiv preprint doi: https://doi.org/10.1101/2024.05.28.596296; this version posted June 2, 2024. The copyright holder for this preprint (which was not certified by peer review) is the author/funder, who has granted bioRxiv a license to display the preprint in perpetuity. It is made available under aCC-BY 4.0 International license.

However, **Figure 10C** contains an additional setting. Here, we replaced the usual NoisyOracles entirely with GaussianOracles. These return samples from a Gaussian distribution regardless of the input. These GaussianOracles are initialized with a very large spread ($\sigma = 10^6$) centered on the mean value of each objective from past experiments. In this setting, sigma far outstrips the dynamic range of the data. We created this experiment to stress the performance degradation from overwhelming noise (or simply uninformative models). This does not mean that we stopped scoring. Rather, this experiment maintains the ratio of 10 molecules scored for every 1 tested, but those scores are not predictive of actual measurements whatsoever.

Figure 10B simply visualizes the inverted survival curves and confidence intervals from **lifelines** (version: 0.27.8), where the duration is the number of molecules that have been made and tested in the campaign.

For the exponential regression in **Figure 10E**, we use the **lifelines** Kaplan-Meier estimator to find costs associated with 2000 linear increments spanning 0.00 to 0.80 success rate. We use this estimator because it takes right-censoring into account. The costs should again be interpreted as the minimum spend necessary to achieve that success rate. By inspection of the cost curves, we proposed a 4-parameter exponential fit:

$$\textbf{cost}\,(\sigma, \text{success rate}) = a \cdot \exp\left(b \cdot \sigma + c \cdot \text{success rate}\right) + d \tag{8}$$

We fit this equation to the costs using `scipy.optimize.curve_fit` in **SciPy** (version: 1.13.0). The fitted parameters were `(16.65396258, 1.07881681, 2.13219256, 18.28661156)`. We again used **SciPy** to find the Pearson correlation coefficient, and we used **NumPy** to find the mean absolute error.

The last panel, **Figure 10F**, asks what would be the marginal benefit of each calculation made by predictive models, in terms of savings from finishing programs sooner. To do so, we compared our model error experiments to the case of no scoring whatsoever. We reason that this is equivalent to the medicinal chemist selecting compounds for synthesis based on their expertise alone. Formalizing this intuition, we found the median dollar cost from 100 replicates with the same experimental settings except that every compound that is ideated is made and tested (scoring models are not used in selections). Using a reported average price of $3000 for compounds that are custom-made by CROs[87], we found the median dollar cost of these trials, which was $1,812,000.00 (604 molecules made).

Across 10,000 bootstrap samples, we found the means and 95% confidence intervals of the difference between that median cost and the corresponding dollar costs for every trial (i.e., the savings), divided by the number of molecules that were scored during the trial. These represent the marginal value gained per calculation. We shaded the region of the chart where the experimental error (the expected replicability of the underlying Oracles sans Gaussian noise). The irreducible experimental error may explain why the marginal value per calculation does not rise as dramatically at the minimal error rate.

Batch size

Here, batch size refers to the number of molecules that participate in an action in each step of the DMTA cycle. The exception is the Score step, which we hard-coded to be 5 times the batch size, chosen to correspond to the number of analogs that were ideated during Design steps. In other words, every compound that is ideated is scored. Since there are two rounds of ideation and scoring in each DMTA cycle, the net effect is that the number of molecules designed and scored is 10 times that of molecules made (80:8). We call this a 10:1 *scoring ratio*. To illustrate the effect of batch size on these numbers: if the batch size were 96 instead of 8, the corresponding number of molecules scored within each Score step would be $96 \cdot 5 = 480$. For these experiments, we use batch sizes based on the size of rows and columns in a typical microwell plate, `(8, 16, 24, 48, 96, 192`. For the same reason, these are typical batch sizes of synthesis orders and experiments.

Figures 9A and **B** represent the same analysis as that of **Figure 10B**, except that the intervention is the batch size of each trial and the model error is fixed at $\sigma = 1.0$. The other difference is in **Figure 9B**, for which the cost of each trial is measured in the number of DMTA cycles (indexing the timestep at which molecules were made), rather than the number of molecules made.

bioRxiv preprint doi: https://doi.org/10.1101/2024.05.28.596296; this version posted June 2, 2024. The copyright holder for this preprint (which was not certified by peer review) is the author/funder, who has granted bioRxiv a license to display the preprint in perpetuity. It is made available under aCC-BY 4.0 International license.

Figure 9C uses the same analysis of expected costs visualized in **Figure 10D**, which finds the Kaplan-Meier estimator costs that correspond to a given success rate. Here, we assign costs, in terms of both the number of molecules made and the DMTA cycles (as drawn in the previous two panels). The final visual stratifies these multidimensional costs across success rates.

Scoring ratio

As described in the previous section, the scoring ratio refers to the relative proportions of molecules scored to those that were tested. Using the standard experimental design with batch size 8, we varied the scoring ratio at intervals between 1 and 20. A scoring ratio of 1 indicates that scores were irrelevant to selection, since every molecule that was scored was tested. The analysis in **Figure 11** was followed as previously described.

Statistics

Cumulative distribution functions are computed with inverted survival regression, with a trial's duration given by the number of molecules that were made or the timestep of the last molecule made. We use **lifelines** (version: 0.27.8) to compute this curve and 68% confidence intervals (via Greenwood's Exponential formula).

Visualizations

Unless noted otherwise, all plots were created using **seaborn** (version: 0.13.2).

Code availability.

The Python code used to produce the results discussed in this paper is distributed open source under MIT license https://github.com/choderalab/drug-gym.

bioRxiv preprint doi: https://doi.org/10.1101/2024.05.28.596296; this version posted June 2, 2024. The copyright holder for this preprint (which was not certified by peer review) is the author/funder, who has granted bioRxiv a license to display the preprint in perpetuity. It is made available under aCC-BY 4.0 International license.

A Supplementary Figures

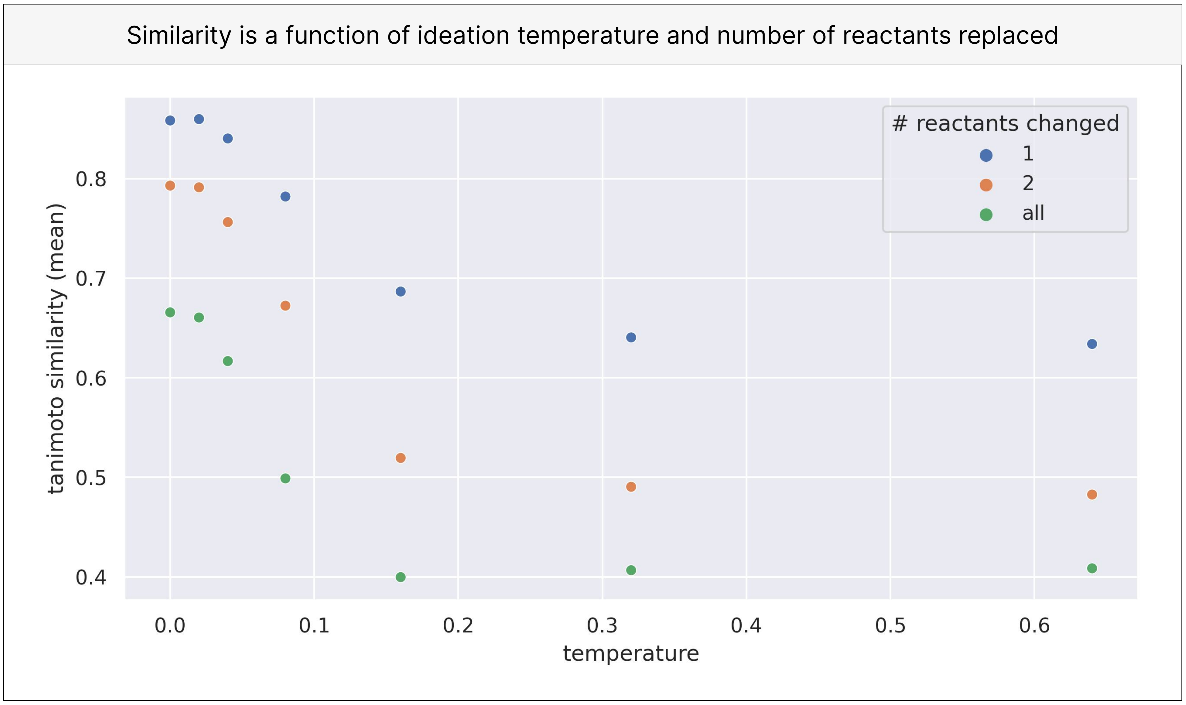

Figure S 12. Tanimoto similarity is a function of ideation temperature and number of reactants replaced. Mean Tanimoto similarity of 500 products (each yielded from a different 2-step reaction) compared to analogs generated with different settings of ideation temperature and numbers of reactants replaced.

bioRxiv preprint doi: https://doi.org/10.1101/2024.05.28.596296; this version posted June 2, 2024. The copyright holder for this preprint (which was not certified by peer review) is the author/funder, who has granted bioRxiv a license to display the preprint in perpetuity. It is made available under aCC-BY 4.0 International license.

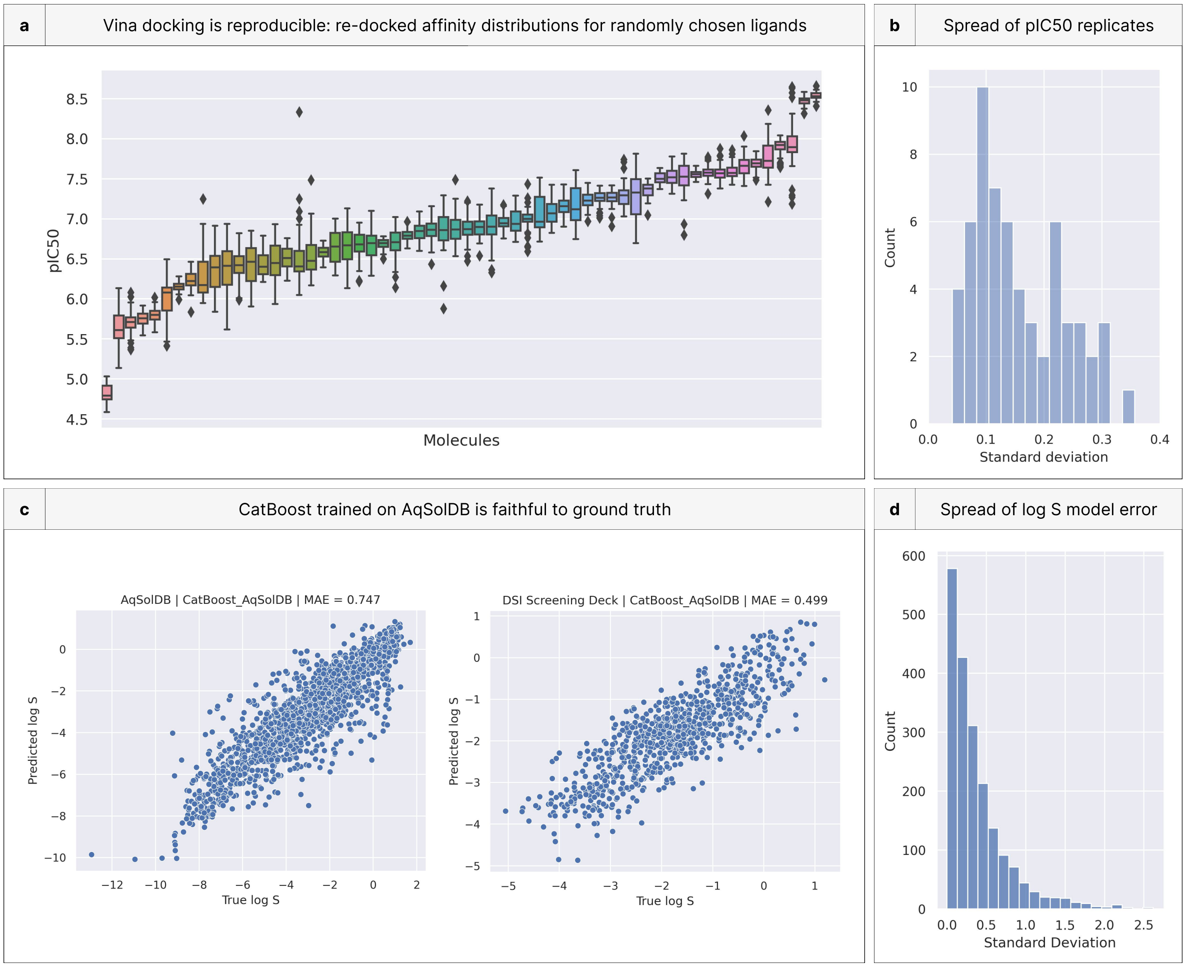

Figure S 13. Scoring models have small but non-zero irreproducibility. (a) Distribution of pIC50 from re-docking 60 ligands (each generated from randomly selected Enamine building blocks). **(b)** The distribution of standard deviations from these ligands. **(c)** Scatterplots of a CatBoost regressor trained on AqSolDB [61], a well-curated solubility dataset, when compared to the held-out test set of AqSolDB and the log S reported for the DSI screening deck. **(d)** Spread of CatBoost model error compared with ground truth.

bioRxiv preprint doi: https://doi.org/10.1101/2024.05.28.596296; this version posted June 2, 2024. The copyright holder for this preprint (which was not certified by peer review) is the author/funder, who has granted bioRxiv a license to display the preprint in perpetuity. It is made available under aCC-BY 4.0 International license.

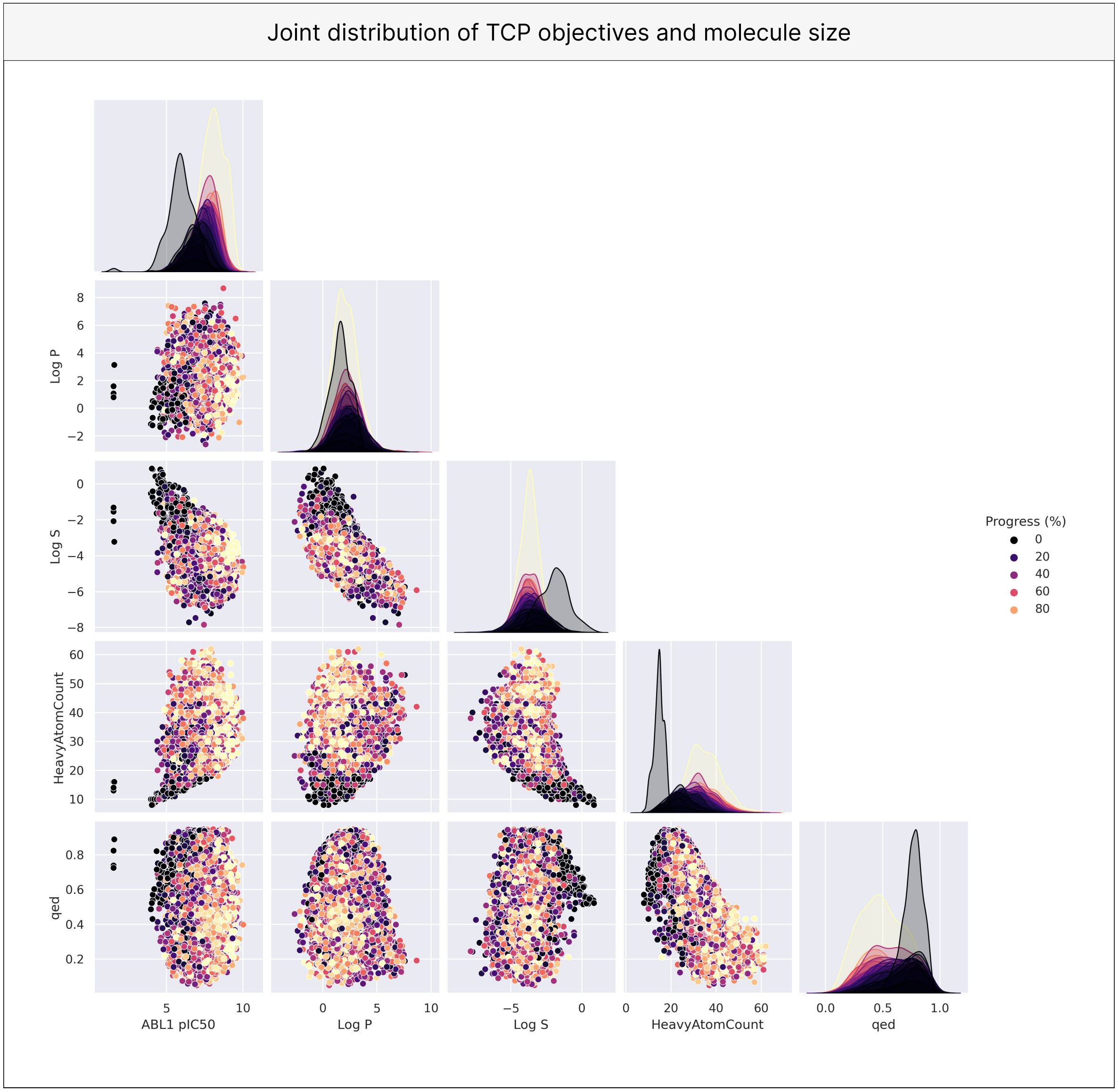

Figure S 14. Joint distributions of various chemical properties across 100 simulated drug discovery campaigns.

bioRxiv preprint doi: https://doi.org/10.1101/2024.05.28.596296; this version posted June 2, 2024. The copyright holder for this preprint (which was not certified by peer review) is the author/funder, who has granted bioRxiv a license to display the preprint in perpetuity. It is made available under aCC-BY 4.0 International license.

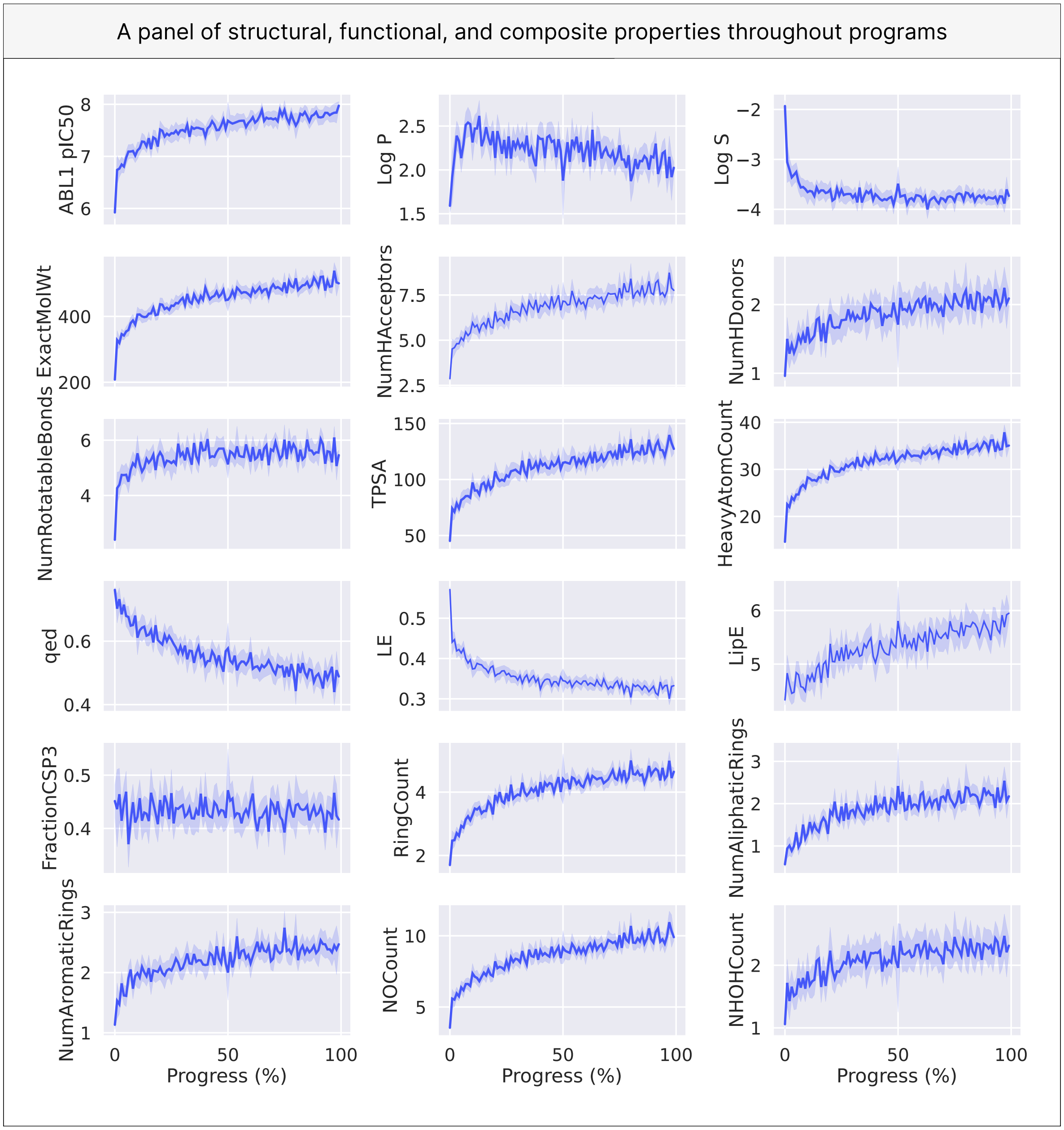

Figure S 15. Scoring models are most useful earlier in discovery programs. Time-dependent averages of chemical properties indicative of drug-likeness averaged over many realizations of the discovery program. The first row represents the TCP objectives. With the exception of LE and LipE, the rest are measured in RDKit (acronyms after the first row, from left to right: NumHAcceptors: number of atoms in the molecule that are hydrogen-acceptors; NumHDonors: number of atoms in the molecule that are hydrogen-donors; TPSA: topological polar surface area; FractionCSP3: the ratio of sp hybridized carbons over total carbons; QED: quantitative estimation of drug-likeness; LE: ligand efficiency, pIC50 divided by the number of heavy atoms; LipE: lipophilic efficiency, difference of pIC50 and Log P; NOCount: number of Nitrogens and Oxygens; NHOHCount: number of N and O atoms with a covalent bond to a hydrogen).

bioRxiv preprint doi: https://doi.org/10.1101/2024.05.28.596296; this version posted June 2, 2024. The copyright holder for this preprint (which was not certified by peer review) is the author/funder, who has granted bioRxiv a license to display the preprint in perpetuity. It is made available under aCC-BY 4.0 International license.

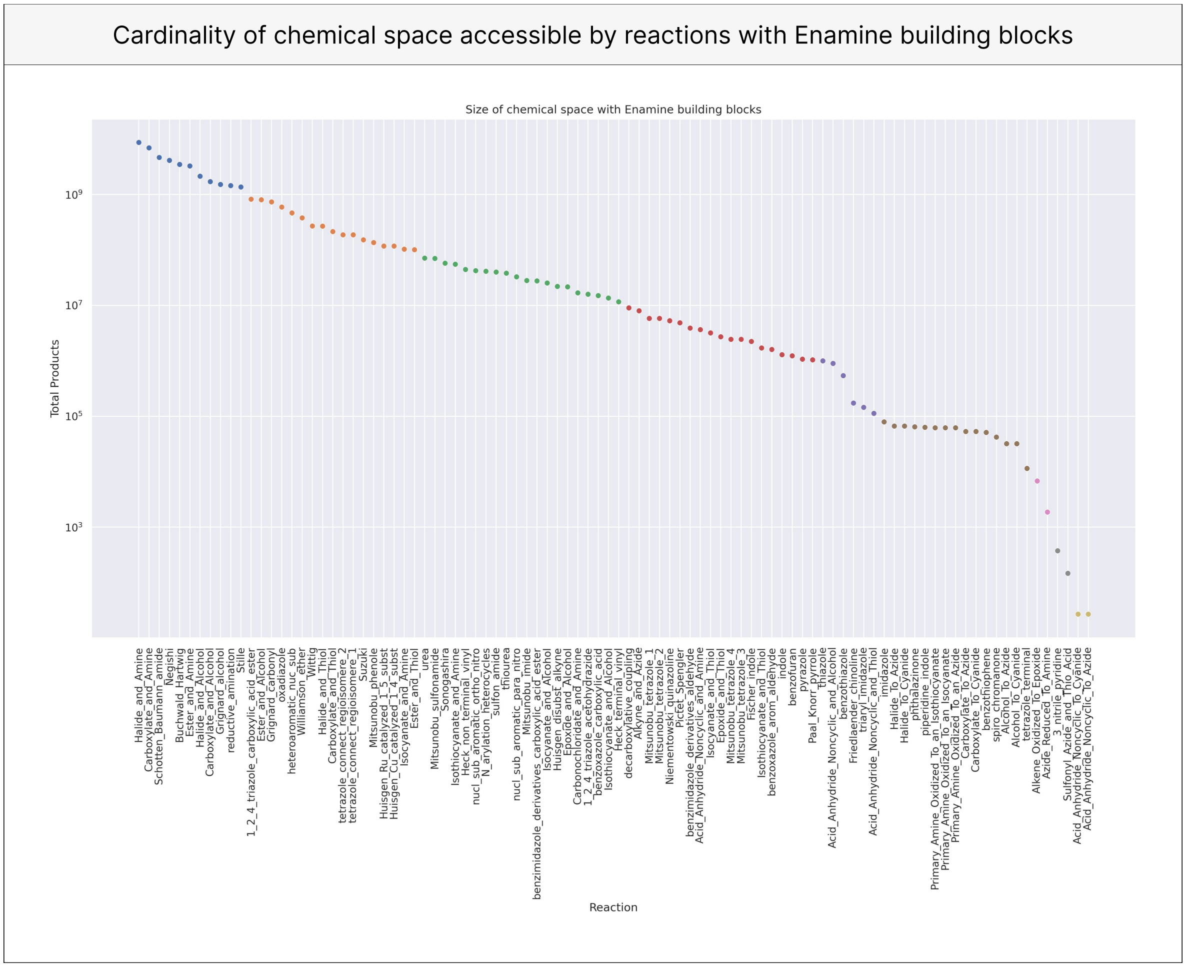

Figure S 16. The accessible chemical space in DrugGym is vast. For each reaction in the SMILESClickChem set, the SMIRKS of its reactants was compared with every building block in the Enamine global stock for substructure match. The product of these values is the maximum possible number of products that can be synthetically enumerated using the reaction and building blocks.

bioRxiv preprint doi: https://doi.org/10.1101/2024.05.28.596296; this version posted June 2, 2024. The copyright holder for this preprint (which was not certified by peer review) is the author/funder, who has granted bioRxiv a license to display the preprint in perpetuity. It is made available under aCC-BY 4.0 International license.

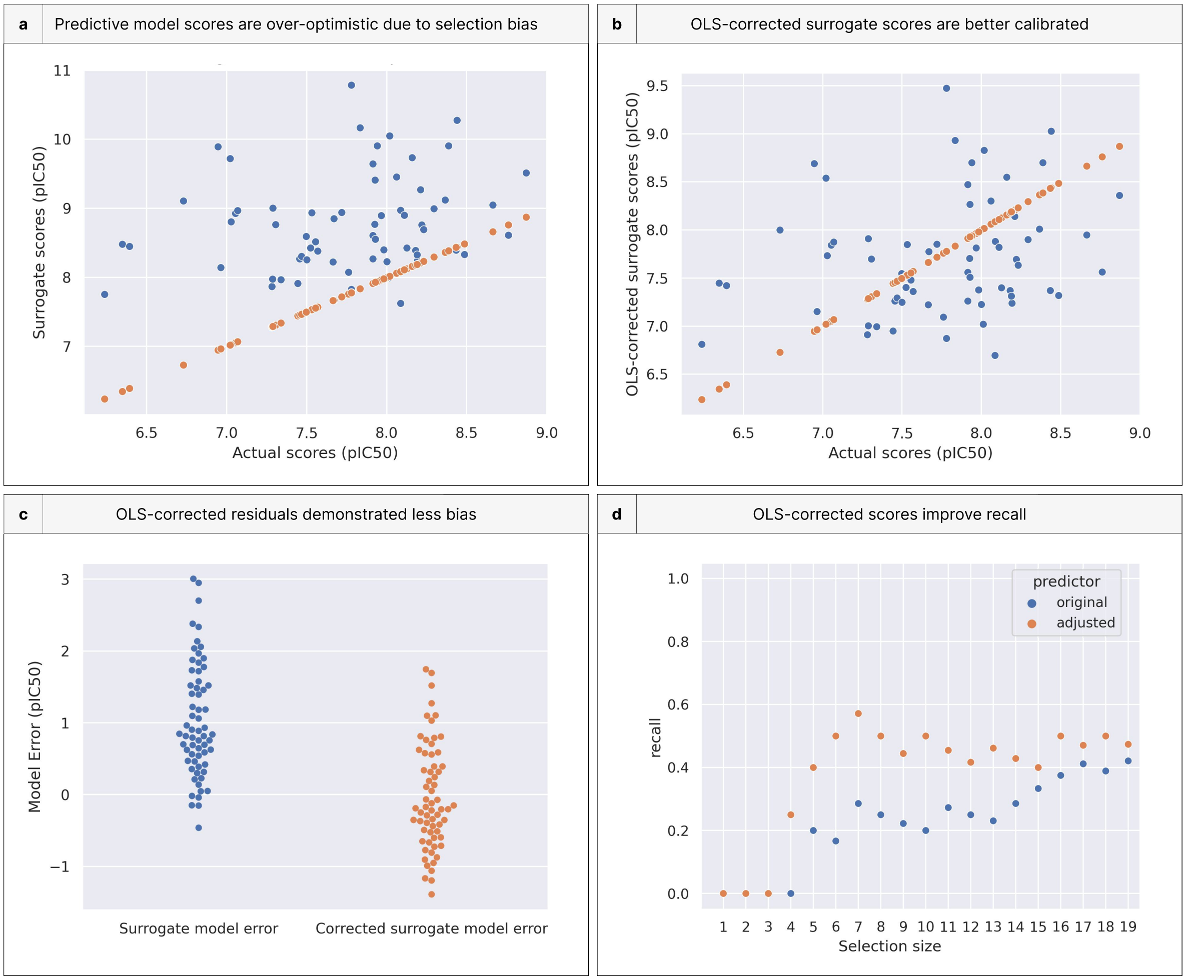

Figure S 17. An OLS correction effectively calibrates predictive model scores. (a) A campaign was simulated as usual with $\sigma = 1.0$. ABL1 pIC50 scores from this campaign were compared to the "true" ABL1 pIC50 Oracle measurements, indicating that selecting for the right tail of noisy scores finds a set of molecules with systematically over-estimated ABL1 pIC50. Orange dots are the actual values and blue are the surrogate scores. **(b)** Using the OLS-adjusted surrogate scores results in visibly better calibration. Orange dots are the actual values and blue are the surrogate scores. **(c)** Compared with the residuals of unadjusted scores, where almost all are positive, the residuals of OLS-adjusted scores are centered on zero. **(d)** The top k molecules as derived from unadjusted and adjusted scores are compared for recall against the "ground truth" top k molecules. OLS-adjusted scores have superior recall.

bioRxiv preprint doi: https://doi.org/10.1101/2024.05.28.596296; this version posted June 2, 2024. The copyright holder for this preprint (which was not certified by peer review) is the author/funder, who has granted bioRxiv a license to display the preprint in perpetuity. It is made available under aCC-BY 4.0 International license.

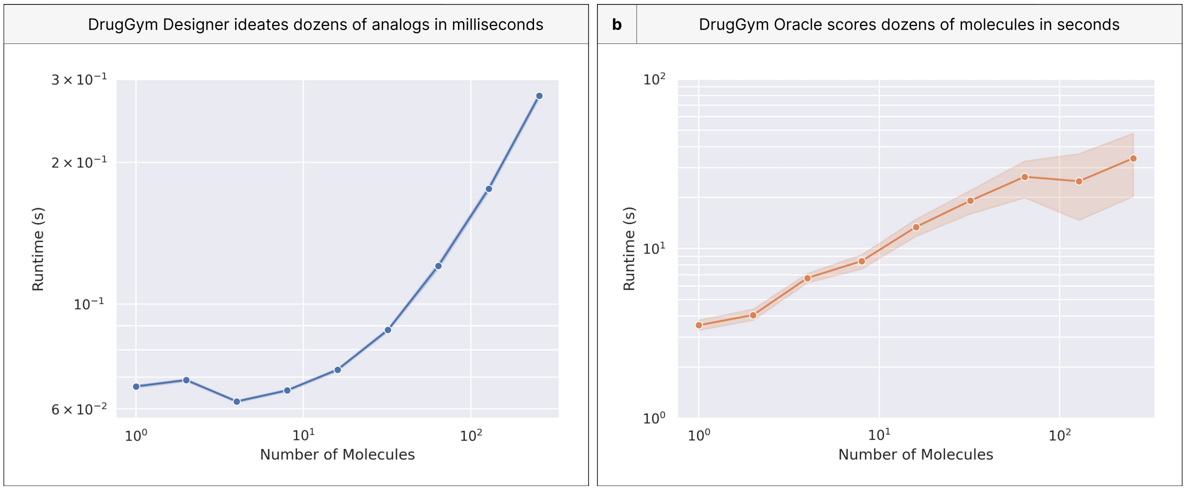

Figure S 18. DrugGym routines run efficiently. Mean and 95% confidence interval for the runtime of various DrugGym routines. **(a)** DrugGym Designer can ideate dozens of analogs in milliseconds. Analogs were designed around a product of two randomly chosen Enamine building block reactants ($n = 1000$). **(b)** DrugGym Oracles can score dozens of molecules in seconds. Analogs generated in the same fashion as in the Designer runtime experiment were scored using DrugGym's standard TCP Oracles: ABL1 pIC50 docking with uni-dock, Log S with CatBoost trained on AqSolDB, and Log P using MolLogP in RDKit ($n = 100$). **System specifications:** 13th Gen Intel(R) Core(TM) i9-13900H 2.60 GHz with 32GB RAM, NVIDIA GeForce RTX 4070 Laptop GPU with CUDA V11.7.64, Ubuntu 22.04.2 LTS.
